# Positive Psychiatry: An Essential Tool to Treat Mental Health in the COVID-19 Era

**DOI:** 10.1192/j.eurpsy.2023.2105

**Published:** 2023-07-19

**Authors:** N. Depa, A. Bocian-Reperowitz, K. Shah, F. Arain

**Affiliations:** 1 Psychiatry; 2 Rutgers New Jersey Medical School, Newark, United States

## Abstract

**Introduction:**

Positive psychiatry is broadly defined as the science of understanding and promoting well-being through interventions that involve positive psychosocial characteristics (PPCs) in people suffering from, or are at high risk of developing mental and physical illnesses (Jeste et al. JCP 2015; 76 675-683). Over the past 3 years, as the pandemic tested the limits of what our minds and bodies can handle, there has been an upward trend in the incidence of mental health conditions, including overdoses, suicide, and substance use (Czeisler et al. MMWR 2020; 69 1049-1057). COVID-19 has highlighted the relationship between the environment and individual mental health, most notably as people have faced increased social isolation, loneliness, and stress (Jeste. SB 2022; 48 533-535). The tools of positive psychiatry can be utilized to further address and target these deteriorations in mental health in hopes of improving outcomes.

**Objectives:**

To educate about the modality of positive psychiatry and how it can be an especially critical tool in treating mental health in the post COVID-19 era. To advocate for the incorporation of positive psychiatry practices into the training curriculum of mental health care providers.

**Methods:**

A literature review was conducted using Google Scholar and Pubmed databases with the term “positive psychiatry” in the title. The search showed 26 results, including 6 systematic reviews and 1 clinical trial. Subsequent searches of “COVID-19” and “mental health” were conducted to create formulations and recommendations for the post COVID-19 era.

**Results:**

Growing evidence shows that PPCs are modifiable constructs that may be associated with improved mental and physical health outcomes. Research during the pandemic has demonstrated that PPCs such as resilience and optimism moderated the trajectory of OCD, depression, and anxiety, and that those with more resilience and optimism displayed lesser decline in their function (Hezel et al. JPR 2022 150 165-172). Beyond mental health, various PPCs, such as social support, have also shown positive outcomes in medical conditions such as hypertension and cardiovascular disease, and ultimately improved well-being (Jeste et al. JCP 2015; 76 675-683).

**Conclusions:**

Based on our literature review, practices of positive psychiatry in conjunction with traditional psychiatry can serve as an invaluable modality in treating patients with various psychiatric conditions and improve mental health outcomes. These positive factors have historically been under-recognized among individuals with or at-risk for mental illnesses. As social distancing, fear of the “unprecedented” and loss of agency became more prevalent over the past couple years, the need for tools to target these notions increases. Further research into optimal incorporation of positive psychiatry into routine clinical practice can help address the trends in mental health brought on by the pandemic.

**Disclosure of Interest:**

None Declared

